# Combination therapy with erythropoietin, magnesium sulfate and hypothermia for hypoxic-ischemic encephalopathy: an open-label pilot study to assess the safety and feasibility

**DOI:** 10.1186/s12887-018-1389-z

**Published:** 2019-01-08

**Authors:** Miho Nonomura, Sayaka Harada, Yuki Asada, Hisako Matsumura, Hiroko Iwami, Yuko Tanaka, Hiroyuki Ichiba

**Affiliations:** Department of Neonatology, Osaka City General Hospital, 2-13-22 Miyakojima-hondori, Miyakojima-ku, Osaka, 534-0021 Japan

**Keywords:** Hypoxic-ischemic encephalopathy, Erythropoietin, Magnesium sulfate, Therapeutic hypothermia

## Abstract

**Background:**

Although therapeutic hypothermia improves the outcome of neonatal hypoxic-ischemic encephalopathy (HIE), its efficacy is still limited. This preliminary study evaluates the safety and feasibility of the combination therapy with erythropoietin (Epo), magnesium sulfate and hypothermia in neonates with HIE.

**Methods:**

A combination therapy with Epo (300 U/kg every other day for 2 weeks), magnesium sulfate (250 mg/kg for 3 days) and hypothermia was started within 6 h of birth in neonates who met the institutional criteria for hypothermia therapy. All patients received continuous infusion of dopamine. Vital signs and adverse events were recorded during the therapy. Short-term and long-term developmental outcomes were also evaluated.

**Results:**

Nine patients were included in the study. The mean age at first intervention was 3.9 h (SD, 0.5). Death, serious adverse events or changes in vital signs likely due to intervention were not observed during hospital care. All nine patients completed the therapy. At the time of hospital discharge, eight patients had established oral feeding and did not require ventilation support. Two patients had abnormal MRI findings. At 18 months of age, eight patients received a follow-up evaluation, and three of them showed signs of severe neurodevelopmental disability.

**Conclusion:**

The combination therapy with 300 U/kg Epo every other day for 2 weeks, 250 mg/kg magnesium sulphate for 3 days and therapeutic hypothermia is feasible in newborn patients with HIE.

**Trial registration:**

ISRCTN33604417retrospectively registered on 14 September 2018.

## Background

Perinatal hypoxic-ischemic encephalopathy (HIE) occurs in 1 to 3% of term or near-term births as a result of hypoxic and/or ischemic insults during labor and delivery [1, 2]. Nearly 20% of affected infants die during the postnatal period, while 25% develop neurologic sequelae [1]. Therapeutic hypothermia in neonates with HIE has been evaluated in six randomized controlled studies and has shown improvements in outcome [3–8]. However, hypothermia was insufficiently effective to avert death or moderate to severe neurodevelopmental disabilities in more than 30% of the patients [3–9]. The addition of other neuroprotective strategies may potentially improve the outcome, but we still do not know which therapy is most effective in combination or whether these therapies are safe.

Erythropoietin (Epo) has various biological roles beyond erythropoiesis. In a preclinical trial using an animal HIE model, Epo has been shown to have both histological and functional neuroprotective benefits [10]. Various doses of Epo have been evaluated in phase I and II studies either alone or in combination with hypothermia therapy [11–15]. These data collectively suggest that Epo is safe and improves neurodevelopmental outcomes. Several phase III trials involving Epo have been proposed or are currently ongoing worldwide [10]. Magnesium, on the other hand, reduces glutamate-mediated excitotoxicity, and is considered as a potential neuroprotective therapy against perinatal hypoxic-ischemic injury [16, 17]. Results of small randomized controlled studies were promising and have shown improvements in neurological outcomes after postnatal magnesium sulfate infusion [18, 19]. Our prospective observational study found that postnatal magnesium sulfate infusion given in combination with dopamine caused no changes in physiologic variables including mean arterial pressure and that deaths and severe sequelae occurred less frequently compared to reported cases of HIE of the same severity [20]. The safety or efficacy of the combination therapy with Epo, magnesium sulfate and hypothermia, however, has not been studied to date. The present study evaluated the safety and feasibility of the combination in newborns with HIE.

## Methods

This prospective single group pilot study was started in January 2014 to evaluate the safety and feasibility of the combination therapy with Epo, magnesium sulfate and therapeutic hypothermia for HIE. The study was approved by the institutional ethics committee, and written parental consent was obtained before enrollment in the study. None of the outcome assessments, including outcomes at 18 months, were blinded.

### Patient selection

Among neonates admitted to the Osaka City General Hospital Neonatal Intensive Care Unit (NICU) and diagnosed with HIE, those meeting our institutional criteria for therapeutic hypothermia were enrolled in the study. The following institutional criteria for hypothermia therapy were developed based on the Japan Working Group Practice Guidelines [21].

A) Infants born at ≥36 weeks’ gestation, admitted to the NICU and meeting at least one of the following criteria: Apgar score of ≤5 at 10 min after birth; continued need for resuscitation, including endotracheal or mask ventilation, at 10 min after birth; acidosis within 60 min of birth (defined as pH of < 7.00 or base deficit of ≥16 mmol/L in umbilical cord blood or any arterial, venous or capillary blood). Infants that meet this criterion are then assessed to determine whether they meet the neurological abnormality criteria (B) by trained personnel.

B) Moderate to severe encephalopathy, consisting of altered state of consciousness (lethargy, stupor or coma) and at least one of the following: hypotonia; abnormal reflexes including oculomotor or papillary abnormalities; absent or weak suck; or clinical seizures. Infants that meet criteria A) and B) are then assessed by amplitude-integrated electroencephalography (aEEG) by trained personnel.

C) At least 30 min duration of aEEG recording that shows moderate (upper margin of > 10 mV and lower margin of < 5 mV) to severe (upper margin of < 10 mV) abnormal background aEEG activity or seizures.

Infants that meet criteria A), B) and C) were enrolled in the study.

Patients who met any of the following exclusion criteria were excluded from the study: infants older than 6 h of birth at the time of initiation of hypothermia therapy; infants with major congenital abnormalities; infants with severe growth restriction with birth weight less than 1800 g; and infants who were considered critically ill and unlikely to benefit from neonatal intensive care by the attending neonatologist.

### Intervention

All interventions were started within 6 h of birth. All patients received whole-body hypothermia therapy at 33.5 °C for 72 h. Epoetin alfa (Kyowa Kirin, Tokyo, Japan) was administered over 2 min intravenously (IV) at 300 U/kg followed by normal saline flush every other day for 2 weeks. Magnesium sulfate (100 mg/1 ml, undiluted solution) was administered at 250 mg/kg intravenously over 2 h for 3 days. All patients received continuous dopamine infusion. The initial infusion rate was 5 μg/kg/min. Followed by increased rate when mean arterial pressure < 45 mmHg.

### Safety monitoring

The safety was assessed throughout the study as described by Wu et al. [13] by monitoring 1) in-hospital death, 2) severe cardiopulmonary collapse during therapy, 3) thrombosis of a major vessel, 4) unexpected events that were likely related to the study treatment, and 5) all other adverse events including liver dysfunction (alanine aminotransferase of > 100 IU/L), thrombocytopenia of < 100,000/μL, persistent pulmonary hypertension of the newborn (PPHN), disseminated intravascular coagulation requiring intervention, sepsis, renal dysfunction (creatinine level of > 1.5), hypertension requiring treatment, and polycythemia requiring treatment. Vital signs such as blood pressure and heart rate were monitored continuously for the first 5 days and every 4 h thereafter until 14 days of age. The Sarnat criteria [22] were used for the evaluation of HIE severity.

### Hospital and neurodevelopmental outcomes

The need for assisted ventilation and establishment of oral feeding at 14 days of age were evaluated as short-term in-hospital outcomes. Brain MRI was also performed at 14 days of age to evaluate brain injury. After hospital discharge, neurodevelopmental disabilities including cerebral palsy (CP), motor delay, cognitive delay, language delay and epilepsy were evaluated by a neurologist on an outpatient basis. The severity of CP was determined based on the Gross Motor Function Classification System (GMFCS). Neurodevelopmental scores at 18 months of age were obtained using a Japanese standardized developmental test, the Kyoto Scale of Psychological Development (KSPD) [23]. Severe neurodevelopmental disability was defined as a KSPD score of < 70 or an abnormal neurologic finding such as hypotonia or hypertonia with functional impairment. Mild neurodevelopmental disability was defined as a KSPD score between 70 and 84, or an abnormal neurologic finding without functional impairment.

### Statistical analyses

Data are expressed as mean (SD), median (range) or n (%). The sample size was determined based on binomial theory to provide evidence regarding the safety level of the death or severe adverse event rate. The exact upper limit with 90% confidence interval for the event rate was defined at 21%. If 9 participants experience 0 death or severe adverse events, the 90% confidence interval is [0, 21%].

## Results

All pre-specified outcomes are described. Participant recruitment started in January 2014 and ended June 2015. Of 9 eligible neonates, all 9 neonates participated. Follow-ups ended in December 2017. Their baseline characteristics are shown in Table 1. The mean 5 min and 10 min Apgar scores were 3.4 and 4.3, respectively. The severity of HIE was moderate in seven cases and severe in two cases. All patients required assisted ventilation, and hypothermia, Epo and magnesium sulfate were initiated within 6 h (mean 3.9 h) of birth.

**Table 1:** Baseline characteristics of 9 patients included in the study

	Mean (SD) or N (%)
Gestational age, weeks	39.7 (2.1)
Birth weight, g	3014 (492)
Caesarean delivery	4 (44)
Outborn	8 (89)
Males	4 (44)
5-min Apgar	3.4 (1.8)
10-min Apgar	4.3 (2.2)
Cord blood pH	6.82 (0.16)
Base deficit, mmol/L	23.3 (6.0)
Moderate encephalopathy	7 (78)
Severe encephalopathy	2 (22)
Seizures on admission	0 (0)
Need for mechanical ventilation	9 (100)
Age at admission, hours	1.7 (1.0)
Age at first intervention^*^, hours	3.9 (0.5)

^*^Hypothermia combined with erythropoietin and magnesium sulfate infusion

**Fig. 1:**
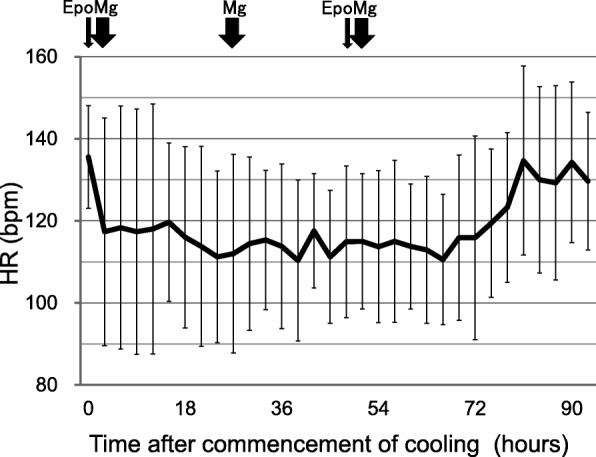
Heart rates during and after hypothermia therapy (mean ± SD). The mean heart rate decreased to 110 bpm during the therapy but rose to 134 bpm after rewarming. HR, heart rate; Epo, erythropoietin; Mg: magnesium sulfate

**Fig. 2:**
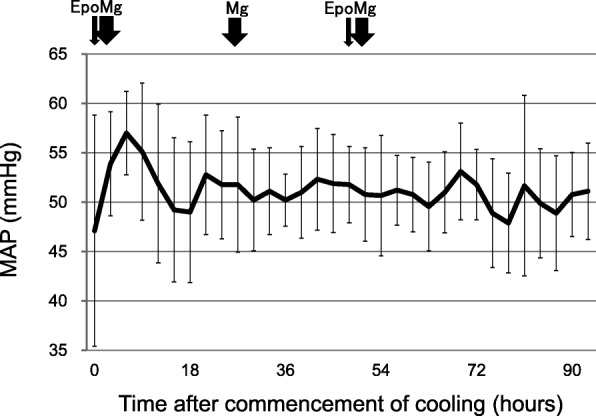
Mean arterial blood pressure during and after hypothermia therapy (mean ± SD). The blood pressure was stable during the therapy. MAP, mean arterial pressure; Epo, erythropoietin; Mg: magnesium sulfate

Death, serious adverse events were not observed (Table 2). PPHN and early onset sepsis were observed in one patient each and were managed uneventfully. The infusion rates of dopamine were between 5.5 and 8.5 μg/kg/min. The mean heart rate decreased from 136 bpm to 110 bpm after cooling and increased to 134 bpm after rewarming (Fig. 1). The mean blood pressure did not change during and after hypothermia (Fig. 2).

**Table 2:** Serious and non-serious adverse events (*N* = 9)

	N (%)
Serious adverse events^*^	0 (0)
Other adverse events	
Liver dysfunction	0 (0)
Thrombocytopenia	0 (0)
PPHN	1 (13)
DIC	0 (0)
Sepsis	1 (13)
Renal dysfunction	0 (0)
Hypertension	0 (0)
Polycythemia	0 (0)

^*^Serious adverse events included death, severe cardiopulmonary collapse, thrombosis of a major vessel, and unexpected events that were likely related to the study treatment. DIC: disseminated intravascular coagulation; PPHN: persistent pulmonary hypertension of the newborn

In-hospital outcomes are summarized in Table 3. All nine patients completed the therapy and survived. Eight of them had established oral feeding and no longer required ventilation support. Assisted ventilation and tube feeding were continued at home in one patient. Abnormal MRI findings were observed in two patients and characterized by diffuse cerebral white matter necrosis and basal ganglia/thalamic necrosis. Post-hoc analysis revealed that 2 patients presented with clinical seizure treated by phenobarbital in the first 72 h.

**Table 3:** Hospital outcomes (*N* = 9)

	Median (range) or N (%)
In-hospital death	0 (0)
Established oral feeding at discharge	8 (89)
Mechanical ventilation at discharge	1 (11)
Normal brain MRI findings	7 (78)
Hospital stay, days	18 (16, 81)

Follow-up evaluations were continued for up to 2 months in one patient and at least 18 months in eight patients (Table 4). Severe neurodevelopmental disability was identified in three patients: CP (GMFCS level V) in two cases and cognitive and language delay (KSPD score of 56) in one patient. These two patients with CP had severe HIE immediately after birth, and showed diffuse cerebral white matter necrosis and basal ganglia/hypothalamus necrosis on their MRI at the time of hospital discharge. The patient with cognitive and language delay had moderate HIE.

**Table 4:** Neurodevelopmental outcomes at 18 months of age (*N* = 8)

	N (%)
Severe neurodevelopmental disability	3 (38)
CP (GMFCS levelV)	2 (25)
Cognitive and language delay	1 (13)
Mild neurodevelopmental disability	0 (0)
Normal neurodevelopmental findings	5 (62)

*CP* cerebral palsy, *GMFCS* Gross Motor Function Classification System

## Discussion

This is the first clinical study to evaluate the feasibility and safety of the combination therapy with Epo, magnesium sulfate and hypothermia for HIE. All nine patients included in the study completed the therapy without developing adverse events likely due to intervention. Death or serious adverse events were not observed. These results suggest that the combination therapy is feasible in newborns with HIE.

Although therapeutic hypothermia has been shown to improve outcomes, deaths or moderate to severe neurodevelopmental disabilities were reported in more than 30% of patients [3–9]. To achieve optimal neuroprotection in hypothermia therapy, other neuroprotective strategies have been investigated, but the safety or efficacy of these therapies has not been established.

Hypothermia, Epo and magnesium sulfate exhibit neuroprotective effects through different mechanisms. The neuroprotective mechanisms of hypothermia include reduced cerebral metabolic rate and energy use, suppression of cytotoxic amino acid and nitric oxide accumulation, inhibition of platelet-activating factor, reduced free radical activity and lipid peroxidation, attenuation of secondary energy failure, and reduced apoptosis and necrosis or brain injury [24]. Epo has anti-apoptotic and anti-inflammatory effects and supports tissue remodeling by promoting neurogenesis, oligodendrogenesis and angiogenesis [10]. The primary neuroprotective mechanism of magnesium seems to be the voltage-dependent non-competitive antagonistic action at N-methyl-D-aspartate receptors [25]. Magnesium may also exert neuroprotective effects through anticonvulsant actions, stabilization of many critical enzymatic reactions, and/or stabilization of the plasma membrane [17, 26]. By combining these mechanisms together, we can expect synergistic neuroprotective effects.

All patients included in the study completed the combination therapy without experiencing any serious adverse events or death. PPHN and sepsis, which are common comorbidities of severe HIE, occurred in one patient each. Hypertension, thrombosis, polycythemia and other adverse events often associated with long-term Epo therapy in adults were not observed. Magnesium infusion can induce hypotension in human neonates. Levene et al. reported that asphyxiated newborn infants given 400 mg of magnesium sulfate infusion over 10–30 min showed risk of hypotension [27]. In the present study, a lower dose (250 mg/kg) was infused more slowly (over 2 h) in combination with dopamine and did not decrease the mean blood pressure. The changes in heart rate during and after hypothermia were similar to those observed in hypothermia therapy alone [28].

The efficacy of Epo for HIE has been evaluated at varying doses between 250 and 2500 U/kg in phase I and II clinical settings [11–15]. At the time of the implementation of the present study in January 2014, the effectiveness and long-term safety of multiple high doses of Epo (1000 U/kg) combined with hypothermia were not yet established. Thus we chose 300 U/kg of Epo given every other day for 2 weeks. The efficacy and safety of combined Epo at 1000 U/kg with magnesium and hypothermia should be considered in future studies.

Our study has limitations. First, it was conducted in a small number of patients without a control treatment and was not designed to evaluate efficacy. However, all nine patients included in the study completed the therapy without developing adverse events. Death or serious adverse events were not observed. Second, as mentioned above we chose the low dose of Epo. Therefore, the safety data will not be applicable to future studies using higher doses.

## Conclusion

The results of this pilot prospective study suggest for the first time that the combination therapy with 300 U/kg Epo every other day for 2 weeks, 250 mg/kg magnesium sulphate for 3 days and therapeutic hypothermia is feasible in newborn patients with HIE. To demonstrate the long-term neuroprotective efficacy and safety of this therapy, phase II and III studies with an adequate sample size are necessary.

## Abbreviations

aEEG: amplitude-integrated electroencephalography
CP: cerebral palsy
Epo: erythropoietin
GMFCS: Gross Motor Function Classification System
HIE: hypoxic-ischemic encephalopathy
KSPD: Kyoto Scale of Psychological Development
NICU: neonatal intensive care unit
PPHN: persistent pulmonary hypertension of the newborn
